# Treatment time for non-surgical endodontic therapy with or without a magnifying loupe

**DOI:** 10.1186/s12903-015-0025-7

**Published:** 2015-03-20

**Authors:** Amy Wai-yee Wong, Xiaofei Zhu, Shinan Zhang, Samantha Ka-yan Li, Chengfei Zhang, Chun-Hung Chu

**Affiliations:** Faculty of Dentistry, The University of Hong Kong, Hong Kong, China; School and Hospital of Stomatology Special Service Clinic, Peking University, Beijing, China

**Keywords:** Endodontic, Time, Magnifying loupe, Clinical trial, Root canal therapy

## Abstract

**Background:**

Use of magnifying loupe may increase the efficiency of dental care. This clinical trial compared the time in performing non-surgical endodontic therapy with or without the use of a magnifying loupe.

**Methods:**

Patients who required primary endodontic treatment in clinical trial centres at the University of Hong Kong (HKU) in Hong Kong and Peking University (PKU) in Beijing were invited to participate in this study. Two HKU dentists and 2 PKU dentists, forming 2 pairs of dentists with similar years of clinical experience, performed endodontic treatments according to the same procedures and used the same materials, either in single or multiple visits. They had no prior experience with the use of a magnifying loupe. One dentist from each pair was trained to use a magnifying loupe (*x*2.5). The treatment time was recorded.

**Results:**

Eighty-four PKU patients with a mean age of 42.8 years and 98 HKU patients with a mean age of 46.0 years were recruited in this study. Ninety-six teeth were treated with a magnifying loupe and 86 teeth were treated without a magnifying loupe. The results showed that treatment time was not associated with age, gender, tooth vitality, or the presence of apical radiolucency or sinus tract. The results of ANCOVA revealed the treatment time was associated with the clinic (HKU or PKU), root canal system (single or multiple), presence of preoperative pain, treatment visit (single or multiple), the use of a magnifying loupe, and the experience of the operator.

**Conclusion:**

In this study, the use of a magnifying loupe could significantly reduce the endodontic treatment time.

**Trial registration:**

Clinical Trials ChiCTR-IOR-15005988 registered 15 February 2015.

## Background

The advancements in the development of dental equipment have enhanced clinicians’ ability and success in performing root canal treatments on their patients. A magnifying loupe is a piece of equipment that increases the efficiency and quality of dental care. Clinicians’ use of a magnifying loupe increases visual acuity [1] and the accuracy of the endodontic procedure. The superior visualisation of the treatment field attained by using a magnifying loupe improves diagnostic capability [1,2] and endodontic outcomes [3]. The use of a loupe also improves the dentist’s working posture and reduces the risk of developing repetitive-stress injuries related to poor body position [4].

Working while using a magnifying loupe has become an increasingly accepted practice in dentistry. Not only has it become an accepted practice for many dental specialties, but is considered mandatory in the endodontic programs at some dental schools [5,6]. With the aid of a magnifying loupe, the visualisation of the surgical field is enhanced; as a result, the diagnostic capability is improved. The magnifying loupe often enables the dentist to confirm the presence of cracks, both in the natural crowns of teeth and in the roots of teeth restored with post crowns [7]. The identification of dentinal cracks was reported to be higher (45%) than the naked eye (39%) in the same study. A careful examination of the gingival margins under the loupe may identify external root resorption. The marginal fit of restorations and the presence of caries can also be checked.

The use of a loupe also increases the efficiency of operative procedures, especially with regard to endodontic and aesthetic dental treatment [1,5,8]. Most dentists have relied principally on their normal visual acuity (20/20 vision). The pulp chamber and even the canals themselves can be sclerosed in teeth with large caries. Even for a dentist who has a good knowledge of canal anatomy can find it difficult to locate the root canals in a calcified pulp chamber [1]. The use of a magnifying loupe may help the clinician to identify the root canal entrance and probable extra canals [3]. Once the canal entrances have been identified, the subsequent cleaning and shaping procedures can be performed in a fairly straightforward manner. A randomised clinical trial concluded that the use of a magnifying loupe may increase the success rate of root-end endodontic treatment [9].

In addition, the use of a magnifying loupe can enhance the accuracy of endodontic procedures [1]. Because of the complexity of the root canal system, canal preparation cannot necessarily be considered complete, even with the help of modern rotary instrumentation and the accompanying irrigation regimes. The magnifying loupe will help the clinician to identify the presence of isthmuses, a C-shaped root canal, and even areas of the canal system that have remained unprepared. The magnifying loupe is also useful for checking canal cleanliness prior to obturation [1]. Hence, the use of a magnifying loupe in endodontic treatment should improve clinical and radiographic outcomes. As Cochrane (2009) found, clinical trial is necessary to substantiate this assertion [10].

Despite the number of advantages of using a magnifying loupe in endodontic treatment, some dentists do not use one during endodontic treatment [1,2,4,8]. Some of them may believe the use of a magnifying loupe increases the amount of time required to perform endodontic procedures [4,8]. Therefore, this study was conducted to compare the time it took to perform endodontic treatment with or without the use of a magnifying loupe. The hypothesis was there would be no difference in the chairside time used in endodontic therapy with or without a magnifying loupe.

## Methods

### Patient recruitment

**Figure 1:**
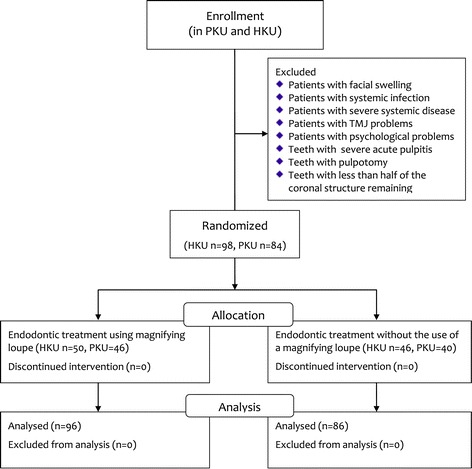
**Flow diagram of the study.**

The study was approved by the Institutional Review Board of the University of Hong Kong/Hospital Authority Hong Kong West Cluster (HKU UW 09–303) in Hong Kong and Peking University (PKU IRB 00001052–10071) in Beijing, China. Chinese patients who were generally healthy and required primary endodontic treatment via the HKU Health Service Dental Clinic in Hong Kong and the PKU School and Hospital of Stomatology Special Service Clinic in Beijing were invited to participate in the study. They were allocated systematically into 2 groups by the receptionists: endodontic treatment with loupes and endodontic treatment without loupes. Teeth with pulpotomies were not accepted, and at least half of the coronal structure had to remain. The protocol of the study was explained to participants and consent was obtained. Patients who had severe acute pulpitis with facial swelling or systemic infection, severe systemic disease, increased stress on the temporomandibular joint musculature, or increased psychological stress were excluded from this study (Figure 1).

### Sample size calculation

The outcome measure is the total chairside time for endodontic treatment. The time for endodontic treatment or root canal therapy (RCT) varies according to the number and shape of the canals. For sample size calculation, we estimated that the time for RCT is, on average, 90.0 minutes; a 13.5-minute (15%) difference between the test and control groups we regarded as clinically significant. The sigma (common standard deviation) was estimated to be 30 minutes. Using an 80% power and a 5% statistical significance level, the number of samples required for each group was 78 or 156 in total.

### Clinical procedure

Two dentists from HKU and 2 dentists from PKU formed 2 pairs of dentists with similar years of clinical experience to carry out the endodontic treatments. One dentist from each clinic was trained to use the same type of Galilean magnifying loupe at 2.5x (N-25R, Yee Mau Industrial Co., Kwai Chung, Hong Kong). The training involved discussion in a seminar and performing endodontic treatment with a magnifying loupe on patients. All 4 dentists performed endodontic treatments on patients randomly assigned by the receptionist with a personal computer. The chairside assistant recorded the total treatment time for each visit to the nearest minute by using a stopwatch.

The dentists received a training workshop prior to this clinical trial to standardise the instrumentation and obturation technique described below. Local anaesthetic was given and a rubber dam was used for isolation. The root canals were prepared by using a crown-down technique, which prepares the coronal part of the canal before the apical portion with rotary endodontic nickel titanium files (ProTaper Ni-Ti files, Dentsply Asia, Hong Kong). A 5.25% sodium hypochlorite solution was used for irrigation. After the preparation and if the time was available, the canal could be obturated at the same visit. If the canals were unable to be completely dried or the scheduled appointment time was used up, the completion of the procedure was scheduled for another appointment. Non-setting calcium hydroxide was used as canal medication and the access of the tooth was sealed with a resin-modified zinc oxide and eugenol cement (IRM, Dentsply Asia, Hong Kong) between visits. The prepared tooth was to be obturated in the subsequent visit.

### Data analysis

The collected data was entered into a personal computer and analysed with the IBM SPSS Statistics 21.0 program. The Kolmogorov-Smirnov normality test was performed for normality testing. Natural logarithmic transformation was applied on the outcome variable (endodontic treatment time) if the data did not follow normal distribution. Two-sample t-tests were used to assess the difference in the transformed treatment time with the independent variables, which were the patients’ gender, study site, quadrant, tooth location, root of the endodontically treated tooth, tooth vitality, presence of apical radiolucency before treatment, sinus tract, preoperative pain, visit of the RCT, and the use of a magnifying loupe. Regressions were used to assess the relationship of the transformed treatment time with the patient’s age and the experience of the operator.

Analysis of covariance (ANCOVA) was used to study the independent variables, including the patients’ gender and age, study site, quadrant, tooth location, root of the RCT tooth, tooth vitality, presence of apical radiolucency before treatment, sinus tract, preoperative pain, visit of the RCT, the use of a magnifying loupe, and the experience of the operator. All of the independent variables were entered into the model. Backward stepwise procedures were performed until only variables demonstrating a statistically significant association remained in the final model. The level of statistical significance was set at 5%.

## Results

A total of 84 PKU patients, aged 42.8 ± 17.3, and 98 HKU patients, aged 46.0 ± 15.5, were recruited (Figure 1). At PKU, 35 teeth with one canal and 49 teeth with multiple canals were treated; at HKU, 38 teeth with one canal and 60 teeth with multiple canals were treated. The endodontic treatment time and variables studied are summarized in Table 1. Also, the patients’ age was not related to the treatment time (p = 0.813), but the experience of the operator was related to the treatment time (p < 0.001) in the regressions (Estimate ± SE is 0.074 ± 0.008, not shown in the table). Because the endodontic treatment time did not follow normal distribution (Kolmogorov-Smirnov normality test, p < 0.001), neutral logarithmic transformation was applied and the transformed endodontic treatment time passed the Kolmogorov-Smirnov normality test (p = 0.200).

**Table 1:** **Endodontic treatment time according to separated variables**

**Group (No. of teeth)**	**Treatment time/min (Mean ± SD)**	**P-value** ^**#**^
Gender		0.408
Male (N = 66)	60.3 ± 30.3	
Female (N = 116)	58.9 ± 34.1	
Study site		<0.001
Hong Kong (N = 98)	76.2 ± 33.5	
Beijing (N = 84)	39.9 ± 17.4	
Quadrant		0.725
Upper (N = 102)	60.8 ± 34.6	
Lower (N = 80)	57.6 ± 30.3	
Tooth location		<0.001
Anterior (N = 42)	36.4 ± 16.7	
Posterior (N = 140)	66.3 ± 33.2	
Root canal		<0.001
Multiple (N = 109)	74.4 ± 31.9	
Single (N = 73)	37.1 ± 17.8	
Tooth vitality		0.985
Yes (N = 75)	62.1 ± 39.3	
No (N = 107)	57.5 ± 27.1	
Apical radiolucency		0.856
Presence (N = 80)	58.5 ± 28.7	
Absence (N = 102)	60.2 ± 35.6	
Sinus tract		0.915
Presence (N = 16)	56.3 ± 24.0	
Absence (N = 166)	59.7 ± 33.5	
Preoperative pain		0.019
Presence (N = 75)	63.7 ± 30.4	
Absence (N = 107)	56.4 ± 34.0	
Visit		0.386
Multiple visits (N = 91)	63.1 ± 37.5	
Single visit (N = 91)	55.7 ± 26.8	
Use of magnifying loupe		0.721
Yes (N = 96)	56.7 ± 23.5	
No (N = 86)	62.5 ± 40.5	

^#^P-value of two-sample *t*-test for log-transformed data.

The results of ANCOVA found that the treatment time was associated with clinic location, root canal, preoperative pain, treatment visit, the use of a loupe, and the experience of the operator (Table 2). The treatment time in Hong Kong’s clinic was longer than that in Beijing (p < 0.001). The treatment time for multiple root canals increased by an additional 85% for a single root canal (p < 0.001). The presence of preoperative pain also increased the treatment time by 9% (p = 0.028). Multiple visits increased the treatment time by 32% more than a single visit (p < 0.001). The use of a magnifying loupe (p < 0.001) and years of the operators’ experience (p < 0.001) helped to reduce the treatment time by 20% and 24%, respectively.

**Table 2:** **Log of endodontic treatment time and variables in the final ANCOVA model**

**Variables**	**Estimate**	**Exp (Estimate)**	**Exp (95% CI)**	**P-value**
Study site Hong Kong Beijing^a^	3.09	21.86	6.15 – 77.69	<0.001
Root canal multiple single^a^	0.62	1.85	1.72 – 2.00	<0.001
Preoperative pain presence absence^a^	0.09	1.09	1.01 – 1.18	0.028
Visit multiple visits single visit^a^	0.28	1.32	1.22 – 1.43	<0.001
Use of magnifying loupe Yes No^a^	−0.22	0.80	0.73 – 0.88	<0.001
Experience of the operator	−0.27	0.76	0.66 – 0.88	<0.001

^a^Reference Category, R^2^ = 0.815, Adjusted R^2^ = 0.810.

## Discussion

According to the results of this study, the null hypothesis that there would be no difference in the chairside time used in endodontic therapy with or without a magnifying loupe is rejected. The belief of some dentists that it takes longer to perform endodontic procedures with a magnifying loupe is not substantiated. There is insufficient control for the potential confounding effect by technical and biological complexity of treatment. For example, if the treatment could not be completed within one visit due to biological reason, the total amount of treatment time would inevitably be longer than those cases completed in a single visit. However, the difference could not be attributable to the use of loupes but was due to biological reasons.

The aim of this study was not to test the effect of training in using magnifying loupes. Otherwise, the study would have recruited many dentists from the two clinics. It is noteworthy that the dentists’ years of dental practice and their experience in using magnifying loupes varied. In this study, only two dentists in each clinic with no experience in using magnifying loupes and with the same years of dental practice were recruited. The small number of dentists recruited could minimise the bias resulting from any unfamiliarity on the part of the participating dentists with the stipulated treatment protocol. Standardisation of treatment protocol and training of the four participating dentists were introduced with the aim of reducing potential bias.

The two dentists who used a magnifying loupe were newly introduced to its use. Learning the magnifying loupe may take some time, but the learning curve is apparently short [1]. The two dentists found the use of a magnifying loupe increased the efficiency of the endodontic treatment and enhanced their accuracy in endodontic procedures.

Another significant benefit of using a magnifying loupe in endodontic treatment is in the field of ergonomics. Endodontic treatment often requires the operator to maintain a certain posture for a considerable length of time, and such a posture can take its toll on the operator. Back, shoulder, and neck problems are common because of incorrect posture [1]. The use of a magnifying loupe enabled the operators to work on patients with an increased working distance. Such a practice also allows an operator to hold his or her back straighter than when working without magnification [1].

This study also demonstrated that the presence of preoperative pain and multiple visits would increase the treatment time. One of the reasons might be that it takes longer to remove the inter-appointment medicament and to dry up the root canal before obturation. Patients who had inflammation might require more time for local anaesthesia, which might even be ineffective in extreme cases.

Another factor that significantly affected the treatment time was the operator’s experience. Experienced clinicians require shorter treatment time than their peers who are less experienced.

This study was performed at two clinical trial centres and allows a comparison between them. The benefits of this two-centre clinical trial are that they allow the study to include an adequate number of participants within 12 months. The different geographic locations also permit the study to include a wider range of patient groups whose tooth anatomy is different.

The results of this study showed that the treatment time was associated with the clinical trial centre, root canal system, presence of preoperative pain, treatment visit, the use of a magnifying loupe, and the experience of the operator. The dentists at the PKU clinical centre were remunerated by the number of items performed, whereas those in the HKU clinical centre were compensated with a fixed salary. This compensation format can explain the difference in treatment time between the two centres. Lo et al. reported that the remuneration system would affect the clinician’s performance [11]. Chu et al. also found that salary-based dentists spent more time communicating with patients than those who were paid by the item (i.e., who tend to perform faster than average dentists) [12].

During data analysis, neutral logarithmic transformation is a common method for manipulating the outlying data from a positively skewed distribution to one that is closer to the bulk of the data to allow the variable to be normally distributed [13]. In this study, using a model in which the outcome variable has been log-transformed and the predictors have not, it is natural to interpret the exponential function of the ANCOVA coefficients. In terms of (simplified) equations, the model with log-transformed outcome variable is $\log \kern0.5em Y={\beta}_{\cap }+{{\displaystyle {\sum}_i^k\beta}}_i{\displaystyle {X}_i}$ for *k* independent variables and thus $Y{=}_e{\displaystyle {\beta}_O}+{{\displaystyle {\sum}_1^k\beta}}_i{\displaystyle {X}_i}$ [14]. Therefore, a 1-unit increase in *X* (an independent variable) will produce an expected increase in log *Y* (outcome variable) of *β* units. In terms of (unlogged) *Y* itself, this means the expected value of *Y* is multiplied by *e*^*β*^. Therefore, while all other variables in the model are held constant, the outcome variable changes by 100 times the exponential function of the model parameter estimate as a percentage for a 1-unit increase in a continuous independent variable. Similarly, to interpret the effect of a categorical independent variable, the outcome variable changes by 100 times the exponential function of the model parameter estimate as a percentage for a specified category compared with the reference category.

In this study, the use of magnifying loupes would help to shorten the time taken to perform non-surgical endodontic treatment. As in other operative procedures, the operators have an improved vision field when performing endodontic procedures. In addition, the improved vision would enhance the clinicians’ confidence in diagnosis before commencing the treatment. The fact that the procedures can be performed with a more accurate visual field may further improve the treatment outcome.

Clinical procedures and diagnostic capability may be better aided with magnification devices in comparison to unaided vision [1]. Dental armamentarium is continuously changing to improve the clinical outcomes in patient care. Human vision has limitations. With the help of magnifying devices, clinicians can view more details and, accordingly, the treatment outcome is enhanced. Therefore, clinicians should incorporate magnifying devices (i.e., magnifying loupes) into daily practice to obtain better magnification [15]. Nevertheless, long-term studies to compare the outcome of endodontic treatment between using operative microscopes and magnifying loupes are still inadequate [10,16,17].

Mamoun reported that aided higher-magnification loupes (4×) may work better than unaided entry-level (2.5×) magnifying devices [5]. Therefore, we may consider that working efficiency is related to the power magnification. Surgical microscopes have higher magnification than magnifying loupes. A study showed that more accessory canals were detected by using a microscope than with a magnifying loupe [18]. Another study also reported that significantly more canal orifices were found using a surgical microscope than by using a magnifying loupe [19]. However, a literature review found there was no significant difference in the treatment results of endodontic microsurgery with respect to the type of magnification device [20,21].

When general dentists encounter difficulty in performing root canal treatment, they refer the case to a specialist. Thus, endondontic specialists are more likely than general dentists to accept challenging cases, and their need to use surgical microscopes is higher. The American Association of Endodontists reported that most of the specialist in the United States incorporate a surgical microscope into their daily practice [22]. On the contrary, it is still not widely accepted by general dentists, for example, in the United Kingdom [23].

Implementing higher magnification will perforce increase the cost of running a clinic and also require a great deal of training on its utilisation within daily practice. Therefore, general dentists still do not widely use higher magnification, so continuing dental education is essential. A study in Scotland revealed that general dentists used magnification primarily in conjunction with crown and bridgework, diagnosis, and radiography to enhance the visual field [4]. In this study, one of the participating dentists thought the use of magnification in clinical practice was time consuming. In addition, clinicians must overcome a steep learning curve to improve their operative work [1]. Therefore, operators face a long journey in the acceptance of the use of magnification in their clinical practice [8]. It also takes time to become accustomed to working with low or high magnification because the depth of one’s field of view is reduced. Furthermore, not all dentists are ready to change their approaches; some of them may consider other factors, such as cost-effectiveness and the training time that would be required [1].

## Conclusion

Endodontic treatment time was significantly reduced by the use of a magnifying loupe.
